# Hospital and Institutional News

**Published:** 1913-10-04

**Authors:** 


### HOSPITAL AND INSTITUTIONAL NEWS.

### "THE HOSPITAL'S" SPECIAL MENTAL HOSPITAL NUMBER.

Next week, as an enlarged issue of The Hospital, we shall publish a Special Number devoted to Mental Hospital questions. Thanks to the co-operation of a series of brilliant contributors the most important questions of the day affecting the present and future of the mental hospital and the status and prospects of its staff will be authoritatively dealt with. "The Mental Deficiency Act" will be considered in a special article by Sir Bryan Donkin, M.D., F.R.C.P., medical adviser to the Prison Commission, whose experience has enabled him to discuss several little understood issues involved. The economic aspect of the Act is of supreme importance, as neither Parliament nor the politicians have apparently been adequately informed of the expense involved in carrying out its provisions on a national scale. Thus other articles will deal with "The Feeble-Minded as Producers," in which the experience gained on this head at Darenth Industrial Colony and elsewhere will be summarised by the authorities most conversant with the problem. The vexed question on the value of existing "Recovery Rates in Mental Hospitals" will be considered by a well-known psychiatrist; while the vital question concerning the relations of "Trade Unionism and Asylum Attendants," showing how far the improved status of the mental hospital staffs has been due to the efforts of their organisations, will be dealt with and a future policy will be outlined. "Government Grants for Mental Research" will also be discussed by Dr. J. Shaw Bolton, medical superintendent of the West Riding Mental Hospital, Wakefield; and Dr. J. Francis Dixon, medical superintendent of the Borough Mental Hospital, Leicester, will contribute an article on "Cooking and Catering at Mental Hospitals." The price of the greatly enlarged Number, which covers practically the whole ground of controversial questions connected with the modern mental hospital, will be, as usual, one penny.

### A PROGRESSIVE FIGHT BY THE CAMBERWELL GUARDIANS.

The efforts of Poor-Law authorities to increase the salaries of the medical staffs at their infirmaries, as we pointed out in our issue of September 20, are often vetoed by the Local Government Board. This has proved to be the case at Camberwell, where the authorities at Whitehall declined to sanction a scale of additional remuneration, but have approved of a modified one. The Board will be able to pay the assistant medical superintendent at the infirmary £200 per year, rising to £250; the second £170, rising to £180; the third £150, rising to £160; and the junior £120. The Guardians, who have been commendably progressive in this direction, have experienced considerable difficulties in filling up vacancies, and it is rare that more than two applicants apply for a post. Recently two ladies attended as candidates for the office of third assistant, but before the Board could ratify the recommendation of the committee to appoint one she had obtained an engagement elsewhere. With regard to the action of the Local Government Board in the foregoing case the Guardians are not going to press for their original increases, but they have determined to urge the Whitehall authorities to agree to their suggestion that the salary of Dr. Keats, the medical superintendent, shall be increased from £475 to £575, instead of to £525 as approved by the Local Government Board. As we remarked in The Hospital a fortnight ago, "No one who is cognisant of the facts in this special case can have any doubt that Dr. Keats has well earned the increase that the Guardians wished to give him." We congratulate the Camberwell Guardians on returning again to the charge.

### THE NEW JOHN COUPLAND HOSPITAL.

By the opening of the John Coupland Hospital last week the people of Gainsborough and the neighbourhood entered into possession of the institution which was left to them by the late Mr. George Coupland, of Hemswell. The present site was purchased in 1908, and Mr. W. Eyre's plans were submitted to Mr. Percy Adams, F.R.I.B.A., the architect of the King's Sanatorium at Midhurst. Building began three years ago. At the opening ceremony Mr. F. Linley, on behalf of the Trustees, said: "Before deciding on the plans Sir Henry Burdett's well-known work was studied and digested. It was decided to adopt the pavilion plan, with administration block in the centre and wards radiating from the angles." After providing a building and keeping it in repair the Trustees were directed to endow it with the residue of the trust, which is expected to produce an income of about £1,200 a year. Patients were admitted from Monday last. The wards consist of one for children with eight cots, a men's ward, and a women's ward, each of which contains ten beds. Terrazzo and oak are used for the floors. The windows are fitted with double-hung sashes, designed to give ventilation without draught, and there are additional windows at each angle. Heating is given by Shoreland's hospital stoves and hot-water radiators. The walls are finished with Robbaliac enamel, and "hit and miss" ventilators are provided over the beds. In the male wing are two rooms which may be used as private wards.

### THE OFFICIAL REPORT ON DOWNS SANATORIUM.

The investigations into the complaints made by patients as to their treatment at the Downs Sanatorium have been embodied in a report which the General Purposes Sub-Committee of the Insurance Committee of the County of London has prepared. The food was alleged to be insufficient and unsatisfactory in quality, the time allowed for visits to patients was stigmatised as grudgingly short, amusements and recreations were said to be too scanty, while too much work, both indoor and outdoor, for the patients was complained of. The sub-committee on the whole vindicate the institution from these charges. Beds, bedding, cooking utensils, and the premises generally are stated to be scrupulously clean; the staff is described as attentive and efficient; the committee were very favourably impressed with the capacity of the medical officer in charge. In The Hospital of August 2 we referred to the investigations of complaints, and, while refraining from comment on the facts *sub judice*, expressed the opinion, which is borne out by the present report, that the commissariat department was almost sure to be free from shortcomings. As regards the commendation of the staff, we are happy to record this testimony to their efficiency, but from the instance which we gave on August 2 it was clear to any experienced sanatorium manager that the management took a wrong tone towards the patients, and, in short, made mistakes which more experienced hands would not have made. We would not press even this point were it not important to remind authorities in season and out of season just now that the administration of a sanatorium requires special gifts and, above all, special experience, which cannot be acquired except by those who have devoted the main part of their lives to the work. It is the non-recognition of this fact which occasions the publication of complaints which experienced administrators would know how to deal with before the public heard of them at all. Another side of this question is discussed in the Editor's Letter-Box this week.

### DUST AND ENTERITIS AT SOUTHWARK INFIRMARY.

The Camberwell Borough Council has unfortunately not adopted the recommendation of its public health committee to request the Local Government Board to hold a public inquiry into the deaths of the nine children from enteritis at Southwark Infirmary, apparently due to dust poisoning from the adjoining refuse siding. The Council has decided instead to instruct its medical officer thoroughly to investigate the whole of the circumstances with a view to discovering the cause of the outbreak, while the surveyor is to report upon the system of dust collection and removal at East Dulwich. It is doubtful whether the Local Government Board, who have suggested to the Council to discontinue the use of the refuse siding, or the Southwark Board of Guardians will be satisfied at the action of the Council. It means considerable delay and, with a recurrence of the hot weather, increased danger to the patients in the infirmary. Neither will they be satisfied with reports of committees prepared by parties who are necessarily anxious to defend their own mode of procedure in dealing with refuse close to an infirmary.

### ATTACK ON THE MEDICAL SUPERINTENDENT.

The debate at the Council meeting unfortunately degenerated into personal attacks on Dr. Bruce, the medical superintendent at the infirmary. At times it was almost unreportable, and even local journals were forced to eliminate a great deal of what was said. It was only natural that the members of the Council would be irritated at an endeavour of a public official to persuade them to abate a nuisance for which they are responsible, but that was no excuse for their attacks on him apart from his public duty. Dr. Bruce has the support of the Local Government Board that the Council's arrangements for dealing with the refuse are unsatisfactory, and at times give rise to a nuisance. This proves conclusively that whether the deaths are due to the close proximity of the refuse or not Dr. Bruce has performed a public service in drawing attention to the nuisance and in attempting to get it removed. The Council was proud of the fact that they had received no complaints from Constance Road Workhouse, also situated close to the refuse siding, and that no children had died there from enteritis in three years. They overlooked the fact that the institution is only used for lying-in cases, and that never, we understand, at any one time more than half a dozen babies are there. They are removed as soon after birth as possible to Gordon Road Workhouse, a considerable distance from the refuse heap. It remains to be seen whether the Local Government Board will allow any further delay.

### SUGAR AS A BUTTER PRESERVATIVE.

An unsuccessful prosecution was brought against a Liverpool firm last week on the charge of having sold butter containing cane or beet sugar. At the request of the Irish exporter from whom the Liverpool firm obtained the butter, his name was substituted for theirs in the summons. He, Mr. E. E. Whitaker, and the City Analyst of Liverpool were the principal witnesses. The latter said that he considered the 1.2 per cent. of cane or beet sugar which he found in the sample of the defendant's butter to be a foreign ingredient. If enough sugar were not used it was not a preservative at all. From 4 to 5 per cent. of salt was necessary as a preservative, and he found 4 per cent. in the sample. The Act said that sucrose, meaning cane or beet sugar could be used. Ten per cent. of sugar might act as a preservative, but a person ought to be content with one preservative. If butter had salt in it, sugar, he considered, should not be inserted at all. Mr. Robert White, an inspector of the Irish Board of Agriculture, and a practical butter-maker, said that sugar was not recognised as a preservative. The defendant stated that sugar was put in because the public at present liked a mild salted butter. There was about 1 per cent. in it. Unless 6 or 7 per cent. was used salt was not sufficient. Sugar was not put in to conceal the taste of the salt. He knew that French butter was cured with sugar. The Stipendiary said he was afraid he must dismiss the summons, as he thought the Act of 1887 contemplated the presence of other preservatives besides salt. He thought that sugar had been proved to be a preservative, and that its presence with salt was not an infringement of the Act. It seems to us inconsistent to say that sugar was put in because the public liked a mild salted butter, and then to deny that sugar was used to cloak the taste of the salt. The question of preservatives is really the question of adulteration, and it is a pity that some means cannot be found for limiting the total percentage of all preservatives or adulterants irrespective of their number. The public then would be protected as far as is possible by the Legislature, and assured that at least a fixed percentage of the substances which it was buying was what any particular substance claimed to be.

### DEATH OF AN INSURANCE ACT RESISTER.

Mr. John Thomas Harthill, medical practitioner, and county magistrate for Staffordshire, has died at his house, Willenhall Manor. Qualifying in 1869, Mr. Harthill studied at St. Bartholomew's Hospital, and became medical officer of health for Willenhall, with which locality his family had been connected for many years. During a smallpox epidemic some years ago he rendered great assistance, and he had been president of the South Staffordshire branch of the British Medical Association. However, in process of time, he was one of those who actively opposed the terms offered to the profession under the Insurance Act. His local position and personal influence will be a loss to his Staffordshire colleagues. He came of a stock that had made independence on honourable terms a tradition, and, as such, was a figure which it will be difficult to replace in the competitive and changing ranks of modern medicine.

### KING'S COLLEGE HOSPITAL CHAPEL.

The chapel at the new King's College Hospital at Denmark Hill was dedicated on September 25 by the Bishop of Southwark, who is the chaplain of the hospital. The service was attended by the Chairman, Lord Hambleden (until recently known as the Hon. W. F. D. Smith), and by members of the committee of management and of the medical staff. Thus one more step has been taken towards the completion of this new hospital, which was formally opened, it will be remembered, by the King and Queen some three months ago. The chapel has seating accommodation for 250 persons: some of the fittings, such as seats, pulpit, and stained-glass windows, have been transferred from the old chapel; and the organ has also come from Portugal Street, but has been enlarged to suit its new functions. An old master, ascribed to Juan Bautista Juanes, has also been transferred from the old chapel to the new. Other fittings of the new chapel have been provided by the generous endeavour of the matron and nursing staff. The bacteriological staff of the hospital have also evidently commenced work at Denmark Hill, to judge by photographs which have been published in the illustrated press, showing the transference of culture tubes from the old laboratories. According to the reporter, great precautions were necessary to avoid a breakage with consequent disastrous results to the inhabitants of Kingsway!

### THE TREATMENT OF ACTON SCHOOL CHILDREN.

The Acton Education Committee is in a dilemma respecting the treatment of children in the Council schools suffering from tonsils and adenoids. An appeal to the local Cottage Hospital some months ago to undertake such work brought a favourable response, but the terms suggested were, in the opinion of the Committee, prohibitive. Regarding further attempt at a local arrangement to be well-nigh impossible, the Committee opened up negotiations with the West London Hospital, and forwarded to that institution a cheque for five guineas, requesting the return of forty out-patients' letters, which would, it was considered, be sufficient to cover all cases of tonsils and adenoids for the period of a year. What has happened in the meantime is not quite clear, though in reply to a question at a meeting of the Committee before the vacation, the Chairman, the Rev. G. S. De Sausmarez, M.A., said "it was a fact that local doctors had been 'getting round' the authorities at the West London Hospital not to do the work at the price." In a letter to the Committee last Thursday, Mr. A. Betteridge, secretary to the hospital, stated that the board had decided that they were unable to undertake the work, and he returned the cheque for £5 5s. forwarded a few months ago. When it is remembered how exacting, on the already crowded out-patient department of a hospital, school work is, it becomes clear that a cheque for £5 5s. is a ludicrously inadequate attempt "to get round the hospital's authorities," which the West London was quite right to refuse.

### MENTAL ATTENDANTS AT CARDIFF.

In a preface to the fifth annual report by Dr. Edwin Goodall, medical superintendent of Cardiff Mental Hospital, the Lord Mayor, Alderman Morgan Thomas, who is chairman of the mental hospital committee, describes the facilities offered by the hospital to the three workers in pathology, apart from Dr. Harold Schölberg. It is interesting to note that the recovery rate for the year is given at 53.5 per cent. for males and 52.5 per cent. for females, when contrasted with the last published figures for mental hospitals as a whole of 28.42 per cent. and 37.21 per cent. respectively. The value of such figures is often debated, and our readers will appreciate the authoritative exposition of the subject which will appear in our Special Mental Hospital Number next week. On the question of research Dr. Goodall emphasises the need for collaboration between workers in different, though allied, branches of investigation. A striking tribute to the attendants at Cardiff is contained in the Report of the Commissioners in Lunacy, who visited the institution last June. "We were very favourably impressed," they say, "by the staff of attendants and nurses, who struck us as being tactful in the management of those under their care, and as being of distinctly superior class." A tribute is also paid to the "very valuable research work" that is being carried on at Cardiff. The attendants must be much gratified at the above reference to their work, which is, even in days when hours of work are being shortened, still extremely arduous, not too well paid, and so largely unrecognised by the public.

### THE EMIGRATION DIFFICULTY.

Dr. Henry Carre, medical superintendent of Woodilee Mental Hospital, near Glasgow, draws attention to the interesting question of the effects of emigration upon his staff. Great difficulty, he says in his report, has been experienced in filling vacant posts, which he considers to be due largely to the popularity of emigration at the present time. Staff difficulties have been caused also by the Insurance Act, which, he says, has caused the greatest discontent among both the men and women members of his staff. He points out that the 3d. or 4d. a week now exacted for medical benefit gives the staffs no more than what they had before. Every class of institution has suffered a similar difficulty since the passing of the Insurance Act, but it would be interesting to hear how far the experience of other hospitals goes to show that emigration is causing a scarcity among mental attendants and nurses. We invite our readers' opinions on this point.

### A VERANDAH MENTAL HOSPITAL.

We have lately been collecting information concerning open-air wards, country hospitals, and so on, and the latest instance which occurs is at the Glasgow District Mental Hospital, Gartlock, where Dr. W. A. Parker, the medical superintendent, has adopted the open-air treatment for almost all his acute cases. "The 'Verandah' Hospital," he writes, "is still the ideal here." Verandahs are provided in connection with the chronic blocks, and convalescent patients practise Swedish drill, and perform farm and garden work. Such verandahs often lead to ingenious devices for prevention of undue heat in summer, and we remember being once shown a "home-made" contrivance for spraying a glass verandah-roof with water, which the medical superintendent of the institution, another mental hospital, pointed out with some degree of pride. There are, however, fashions in medicine and in therapeutics as everywhere else, and a sanatorium superintendent in an article in this issue says that the open-air treatment is being overdone. To this point we shall return in a future article, merely now remarking on the increasing popularity of the verandah as an institutional adjunct in the modern hospital.

### A NEW WILTSHIRE SANATORIUM.

The Dowager Countess of Pembroke, it is announced, intends to build a sanatorium near Wilton, on the Wiltshire Downs, for the benefit of the neighbourhood, in memory of her husband, who died in Rome last Spring. The news was made public at a meeting which the Mayor of Wilton had convened to consider the question of raising a public memorial to the late Lord Pembroke. A letter from the Hon. George Herbert was read, in which the writer stated that Lady Pembroke did not "wish money wasted on a statue." A committee was appointed to confer with the intending donor, and to report at a later date. If we may look ahead, as institutional workers and managers are bound to do, the warning may be repeated in advance: "Whatever building and plans you have, do not fail to appoint as medical superintendent a medical man who has had a special training in the institutional treatment and administration of tuberculous patients."

### THE DRUG HABIT IN FRANCE.

In English eyes the drug habit has always had its home in France, and amateurs of letters still recall the early 'nineties in which young French poets, like Laforgue and also the great Verlaine, used to indulge in absinthe in order to gain inspiration for their masterpieces. Last week the Paris correspondent of the *Times* announced discoveries by the police of a luxurious opium smoking establishment at Vésinet, a Paris suburb, which revelations are stated to show has been frequented by "many well-known people." Whether this will lead to the affair being hushed up, or whether French journalists are preparing for a *frisson*, remains to be seen. But public opinion of late has been awakened by the frequent deaths from cocaine, opium, and ether. The discoveries of new synthesised products makes the task of legislation in coping with the habit a very difficult one, and in England the drug habit, piously disguised as ether tonics, and the like, has several respectable disguises. Mlle. Fleury, whose death led to the discovery of the establishment which she frequented, appears to have been an incurable victim to the habit, which is generally fashionable among the wealthier *demi-monde*, and one of the worst evils of which is its association with practices of a calculated vicious kind. Three of the women who occupied the villa, writes the same correspondent, are to be prosecuted for culpable homicide. Even if convicted the habit is only likely to be driven into another of its protean forms, for legislation is necessarily always behind the fashions of drug *habitués*.

### MEDICAL OFFICERS AND THE COST OF DISPENSING.

It is not often that a request for a reduction of salary is made, and still more seldom, we suppose, does the request lead to increased fees being granted. However, at a recent meeting of the Scarborough Guardians when, on the motion of Mr. W. C. Sawerby, chairman of the finance committee, the novel request was made and the generous response was given. Dr. Vassalli, medical officer for the West District of Scarborough, has held this post at a salary of £45 a year, out of which he had also to provide the medicine. He therefore applied to the Board to take upon themselves the cost of providing and dispensing the drugs, in return for which he asked them to make a proportionate reduction in his salary. The committee did not see its way to alter the conditions of the appointments, of which Dr. Vassalli's was only one, but were convinced of the inadequacy of his remuneration, and it was decided to increase it from £45 to £65 per annum, the conditions of dispensing remaining the same. No better instance could be found of the way in which public authorities inwardly realise how costly does dispensing in a medical officer's work become, than their present refusal to undertake it; and, it may be added, that the Scarborough committee has judged the medical officer's responsibility for drugs worth escaping even at a cost of £20 more per annum. We trust that medical officers and Boards of Guardians elsewhere will take the hint. The tact of the medical officer in presenting his case and the generous reception accorded to it by the Guardians might also with advantage be copied elsewhere.

### THE CENTRALISATION OF POST-GRADUATION TEACHING.

At the opening of the medical schools it is opportune to remember that post-graduation medical teaching also demands a share of the attention which at this time is largely devoted to undergraduate work. In this connection attention should be directed on a recent letter of Dr. C. O. Hawthorne to the Glasgow newspapers, in which he condemns "rival schemes in association with the various hospitals" as foredooming to failure the Glasgow School. He sums up his own views in these incisive words: "There can be no doubt that in the immediate future post-graduation opportunities for practitioners . . . will be demanded and utilised to an increasing extent . . . and all that is needed is the co-ordination of existing agencies." Dr. Hawthorne therefore urges the adoption of some central organisation and control of the schemes for post-graduation—a term which on literal grounds he prefers to the inaccurate crasis "post-graduate." He ridicules those hospitals which have announced that their laboratories are arranged for post-graduates, a tautological inexactitude which deserves, though it may survive, the criticism that Dr. Hawthorne launches upon it. Once a graduate always a graduate, but there is a graduation beyond, as well as before, the taking of a medical degree. If Glasgow can show the way in which post-graduation teaching can be centralised it will have accomplished something which will claim from medical schools everywhere the closest attention.

### A DOCTOR AND DEATH CERTIFICATES.

A medical practitioner, Mr. Michael Joseph Ryan, and an assurance superintendent, described as formerly a town councillor, were committed at Bolton to the Manchester Assizes last week on charges of obtaining some £500 by false pretences and obtaining and using false certificates of death. The prosecution alleged that the assurance superintendent obtained false death certificates from the accused practitioner, thus enabling the former to obtain payments from the assurance company of claims on the policies of persons still alive. The practitioner's defence was that the certificates were not intended for official registration, but were meant to be used for the company's purposes. The Chairman of the Bench said that the case raised important questions, and demanded strict investigation, particularly regarding the receipt of benefit by persons without insurable interests. Heavy bail was fixed for the two defendants mentioned. Without commenting on the present case, it is noteworthy that the issues customarily raised when alleged false death certificates are the object of inquiry are absent here. The result of the case, whatever effect it may have upon those immediately concerned, however, may be to concentrate attention upon the ease with which such certificates are often accepted by local authorities, particularly in the North, and to the need for making more stringent regulations concerning them.

### THE ALMONER SYSTEM AND THE HOSPITAL SATURDAY FUND.

At the quarterly meeting of the Board of Delegates of the Hospital Saturday Fund, to be held at 34 Red Lion Square, W.C., on the 4th inst., the following resolution will be proposed by Mr. D. Kennedy:—"That the Board of Delegates, having learnt that the managing bodies of many of the Metropolitan hospitals desire to retain the right of inquiry into all cases presenting themselves for treatment, whether recommended by the Hospital Saturday Fund or any other subscriber; and only being desirous of preventing any abuse of the Hospital Almoners' System, request the Distribution Committee of the Fund to assess the awards for 1913 in the usual way. At the same time, if in any case the hospital authorities consider that the recommendation of the Fund is being abused, the Board request that the case should be brought to the notice of the Secretary of the Fund, who will cause inquiries to be made and report the result to the Distribution Committee." This resolution is evidently a sincere attempt to get over the difficulties which have been created by an agitation fomented against the almoner system, which rested, we gather, largely on a misapprehension. If the resolution is carried the Distribution Committee of the Hospital Saturday Fund will continue to work on the basis which has proved satisfactory in the past so far as the hospitals are concerned. We feel confident that the managers of the participating hospitals will have no difficulty in agreeing to instruct each almoner to bring to the notice of the Hospital Saturday Fund each case where the hospital authorities consider that the recommendation of the Fund is being abused. We congratulate those responsible for the resolution on the statesmanlike wisdom they have exhibited in formulating the present solution of a question which has engaged the attention of the delegates for some time past.

### SATURDAY FUND AND FAMILY COLLECTIONS.

Hospital Saturday this year will be held on October 11, the date, by the way, on which the special—we might almost say gala—performance will be held at the Coliseum on behalf of the Charing Cross and French Hospitals. In an appeal which Mr. A. W. Davis is issuing, it is stated that the income of the Saturday Fund is suffering at the rate of £100 a week, and, if the total of these weekly deficiencies is to be made good, a special effort is required. The expedient suggested is family collections, and sheets for recording them may be obtained from Mr. A. W. Davis at 54 Gray's Inn Road. We shall be curious to learn the success of this attempt, which, if it "catches" at all, might be highly successful. Members of individual families have means of bringing pressure to bear upon one another, which are unknown to the most importunate of official collectors, and from which, did they know them, such officers might perhaps shrink! The invocation of the near relation may yet retrieve the income of the Fund.

### THE CENTRALISATION OF SEROLOGY.

In a quiet part of Copenhagen stands the Serum Institute of Denmark, be it noted, not the serum institute of the Danish metropolis, but the headquarters for the whole kingdom. The institute is almost picturesque in its situation and resembles nothing so much as the country house of some well-to-do citizen, with its lodge, its stables, and gardens. In its exceeedingly well-equipped laboratories many distinguished foreigners have worked in recent years, to name Noguchi for one. But the point we wish to call attention to is that here in this central institution all the Wassermann tests are done for the whole of the Danish medical profession. Bacteriologists will appreciate what this means. It means that this highly complicated reaction is done not only by an expert, but under conditions which eliminate the personal factor almost entirely. The results, therefore, are strictly comparable and may be confidently standardised. Naturally the technique is of the most highly developed order and yet simplified by reason of the practically constant flow of work. The fees charged to hospitals and ordinary patients are exceedingly low, and if desirable remitted altogether. Three to five shillings represents the fee paid by the majority of the cases. In the grounds of the institute there is quite a menagerie, for large numbers of goats, monkeys, guinea-pigs, and a few horses are kept for inoculation experiments and antitoxin preparation. A certain amount of clinical bacteriological work is, of course, done in laboratories attached to the several hospitals in Copenhagen, but research work and nearly all the serological investigations are carried out in this Serum Institute, so ably controlled by Dr. Mahdsen.

### A VACANCY AT THE MIDDLESEX HOSPITAL.

It is announced that a vacancy exists for a dental surgeon to the Middlesex Hospital through the resignation of Mr. W. S. Nowell, M.A., M.R.C.S., L.D.S. A Middlesex Hospital man himself, Mr. Nowell is still in the prime of life, for his dental qualification dates from 1895 and his medical one from two years later. He has, however, done a great deal of hard work during his tenure of the appointment, and has proved himself a worthy member of the exceptionally excellent staff which this hospital has possessed in late years. The advertisement soliciting applications for the vacancy requires that candidates must be registered medical practitioners as well as licentiates in dental surgery of the Royal College of Surgeons of England. This requirement is the usual condition of these posts at general hospitals, and quite rightly, for the dental surgeon, much more than in special dental hospitals or in private practice, is called upon to advise and to execute dental surgery in those already suffering from other diseases, frequently serious. It is, therefore, important that he should have a general knowledge of medicine and surgery more extensive than that which is required for the licentiateship in dental surgery alone.

### RE-OPENING OF THE SCHOOL OF PHARMACY.

In the Pharmaceutical Society's School of Pharmacy, in Bloomsbury Square, which entered upon its seventy-second year on October 1, many hospital pharmacists who have achieved distinction in their calling have recived their technical education. The re-opening of the old school is, therefore, a matter of interest to them. In 1841, when the school was established, there was nothing of the kind in this country, and there were only one or two institutions of a similar nature on the Continent. The inaugural address on Wednesday was delivered by Dr. F. B. Power, who chose as his theme "The Influence and Development of the Researches of Daniel Hanbury"; he held up Hanbury, who was a student in the school in 1844, and a brilliant research worker, as an example for present-day students of pharmacy to follow, and he encouraged young pharmacists to explore in the great field of research which was opened up by Hanbury. "The vast Continents of Africa and Asia, and the forests of South America, as well as the islands of the sea, doubtless contain among their natural resources," said Dr. Power, "many thousands of plants which have never yet been touched by human hand. . . . There can be no doubt that the chemical study of the countless number of plants whose constituents are, as yet, completely unknown, will not only reveal much of scientific interest, but also principles of medicinal value." During the proceedings at Wednesday's meeting Mr. E. White, president of the Pharmaceutical Society, who was in the chair, presented the Hanbury Medal to Dr. Power. The medal is awarded biennially for high excellence in the prosecution of original research in the chemistry and natural history of drugs, the object being to perpetuate the memory of Daniel Hanbury.

### THIS WEEK'S DRUG MARKET.

There has been a fair consumptive demand in the Drug market this week, but special features of interest have been few. The advancing tendency of prices which was so noticeable a short time ago is by no means pronounced at present; in fact, the tendency is rather in the other direction. Menthol is again cheaper, and there is an impression that prices may decline still further; not very long ago its value was more than double the present figure, while, on the other hand, within easy recollection it was at one time only a third to-day's price. Buyers of menthol should act cautiously, as the market is a very peculiar one. High prices are still quoted for oil of almonds, and any immediate relaxation is not expected. The price of oil of cloves has been substantially reduced in consequence of the fall in the value of cloves. Cream of tartar is slightly dearer. There is little change in the Opium market, but if anything prices are slightly weaker. Quinine is unaltered in price. The Glycerine market is being carefully watched by buyers, and an advance in price would not come altogether as a surprise. Citric acid still tends dearer. Oil of aniseed is slightly lower in price. In a few weeks the Drug market will in all probability be more active, and already inquiries are more numerous.

